# Factors associated with the revolving door phenomenon in patients with schizophrenia: results from an acute psychiatric hospital in Romania

**DOI:** 10.3389/fpsyt.2024.1496750

**Published:** 2025-01-24

**Authors:** Vlad Dionisie, Maria Gabriela Puiu, Mihnea Costin Manea, Emanuel Moisa, Ana Maria Dumitru, Leila Ibadula, Aliss Madalina Mares, Corina Ioana Varlam, Mirela Manea

**Affiliations:** ^1^ Department of Psychiatry and Psychology, “Carol Davila” University of Medicine and Pharmacy, Bucharest, Romania; ^2^ Department of Adult Psychiatry, “Prof. Dr. Alexandru Obregia” Clinical Hospital of Psychiatry, Bucharest, Romania; ^3^ Department of Anaesthesia and Intensive Care Medicine, “Carol Davila” University of Medicine and Pharmacy, Bucharest, Romania; ^4^ Reteaua Medicala Victoria, Bucharest, Romania

**Keywords:** schizophrenia, revolving door, frequent hospitalization, inpatient mental health service, substance use disorder, aggression

## Abstract

**Background:**

The revolving door phenomenon refers to patients with frequent hospital admissions and emerged after deinstitutionalization reforms have been implemented. Schizophrenia is a severe and debilitating mental disorder and has frequently been identified as one of the most prevalent disorders among revolving door patients. Therefore, this research aimed to identify socio-demographic and clinical factors associated with the revolving door phenomenon in patients with schizophrenia.

**Methods:**

We conducted an observational and retrospective cohort study and collected data from the medical records of individuals admitted to the largest psychiatric hospital in Romania during a 2-year period. Patients with three or more admissions during a 12-month period were identified as revolving door.

**Results:**

Of the total of 635 patients included in this study, 108 met the criteria for revolving door. Patients had a mean age of 44.55±12.83 years and most of them were single (81.7%) and receiving a disability pension (68.7%) and had an illness duration of more than 5 years (81.9%). Male gender (p=0.000), younger age (p<0.05), presence of psychiatric comorbidity (p<0.05), substance use disorder (p=0.000) and alcohol use disorder (p<0.01) were associated with the revolving door patients. A binary linear logistic regression revealed that male gender (OR=1.92, 95%CI:1.21-3.08), shorter hospitalization (OR=0.982, 95%CI:0.964-1.000), substance use disorder (OR=2.47, 95%CI:1.16-5.26), verbal (OR=1.44, 95%CI:1.05-1.98) and physical (OR=1.331, 95%CI:1.017-1.744) aggression were predictive factors for frequent use of inpatient services.

**Conclusions:**

The results may facilitate development of future reform policies aimed at reducing the revolving door phenomenon, including implementing transitional care interventions between hospital and community services.

## 1 Introduction

After the fall of the communist regime in 1989, Romanian authorities followed the international trend of deinstitutionalization of psychiatric care and initiated a reform of the public mental health system that included a drastic reduction in the number of beds in psychiatric hospitals by 15.7% between 1990 and 2018 (1). In turn, the authorities set up a network of mental health centers as alternative community-based services to shift care provision from hospital to outpatient and community care (2). Unfortunately, the lack of adequate funding, strong coordination between community-based services and hospital services and integration of other health and social services with the psychiatric system led to a poor development of community services in Romania in comparison with other European countries (3, 4). Similar scenarios have been noted in different countries all over the world, despite the differences in the organization of the mental health care systems (5–7). The reduction of hospital beds has resulted in limiting length of stay and premature discharge to ensure access to inpatient care for as many severely ill patients as possible (5, 8). Therefore, the deinstitutionalization policy coupled with inadequate alternative services in the community set the stage for the revolving door phenomenon which refers to a specific group of patients with a pattern of multiple readmissions over a relatively short period of time.

The revolving door phenomenon was researched early on because of its far-reaching implications. More precisely, it puts a financial strain on mental health systems since a small number of patients (<10%) are allocated large amounts of healthcare resources (20-30%) (6, 9). In addition, re-admission is seen as “therapeutic failure” and prevents other patients to receive inpatient care (6, 8). Despite extensive research on this subject, there is no agreement on the criteria used to conceptualize the phenomenon. Typically, researchers have used combinations of different numbers of hospitalizations in different time periods. Botha et al. (2010) included in the proposed criteria the total number of days spent in hospital and whether the patient was treated with clozapine (5). The most frequently applied criteria for revolving door patients are three or more hospitalizations in one year (6–8, 10, 11). A recent systematic review conducted by Fonseca Barbosa et al. (2023) concluded that revolving door patients had a mean (SD) number of admissions/year in the analyzed studies of 1.72 (0.85) (12).

Revolving door phenomenon is still not clearly understood but is considered to have miscellaneous causes (i.e. personal, clinical, environmental, and psychiatric system organization characteristics) (10). Current research identified several socio-demographic factors associated with frequent service users, such as younger age, male gender, being single, lower educational level and unemployment or receiving disability pension (6, 8, 11, 13–17). Schizophrenia, personality disorders, alcohol or substance use disorders, particularly cannabinoid use, were observed as clinical predictors of the revolving door phenomenon. Other clinical correlates include an earlier age of illness onset, aggressiveness and violence, treatment non-compliance, and long-acting injectable (LAI) antipsychotic (6, 8, 11, 15–17).

Although there is a large body of evidence on the revolving door phenomenon in the general psychiatric population, less is known about it in patients with schizophrenia. The scarce research conducted until now on this patient group have showed that younger age, male gender, not being in a relationship, and alcohol or other psychoactive substances use disorder are associated with the revolving door phenomenon (18–21). Schizophrenia is a severe mental disorder with slightly reduced prevalence and incidence since 1990, though its disease-associated burden remains unchanged (22). Starting in early adulthood, it imposes high healthcare costs (23). Research into hospital access and care patterns for these patients is therefore crucial.

Lack of real-world data regarding the mental health sector is a shared characteristic of eastern European countries, which has prompted others to name the region “a blind spot on the mental health map” (24). Reliable data is highly needed for the development of effective future public health reform policies.

To address all these gaps, this study set out to determine the socio-demographic and clinical factors associated with the revolving door phenomenon in patients with schizophrenia addressing the “Prof. Dr. Alexandru Obregia” Clinical Hospital of Psychiatry. Also, this study was aimed at developing a prediction model for the revolving door phenomenon. Practically, insights from this research could contribute to enabling tailored intervention to reduce relapse rates and enhance recovery. Also, the results could inform better resource utilization and continuity of care local policies. Because the revolving door phenomenon is a global phenomenon, this research could fill in knowledge gaps across diverse healthcare systems and contribute to evidence-based approaches in psychiatry.

## 2 Materials and methods

### 2.1 Study design and population

This study is an observational and retrospective clinical cohort study that investigated the revolving door phenomenon in “Prof. Dr. Alexandru Obregia” Clinical Hospital of Psychiatry, Bucharest (Romania). The study received approval from the local Institutional Ethics Committee (approval no. 113/09.02.2023) and followed the ethical principles of the Declaration of Helsinki.

“Prof. Dr. Alexandru Obregia” Clinical Hospital of Psychiatry is the largest psychiatric service in Romania, offering both inpatient and outpatient care, including involuntary admission. The hospital serves patients from Bucharest, Romania’s capital city (with a population of almost 2 million individuals), and surrounding counties and has a near-monopoly regarding inpatient services in its catchment area. The emergency department is opened 24 h, seven days a week, and offers psychiatric care for all addressing patients.

Only adult patients (≥18 years old at the time of the first admission during study period) admitted during 1 January 2021 and 31 December 2022 and discharged with a primary diagnosis of schizophrenia according to the International Classification of Disease, the 10th revision, (F20) were included in this analysis. Patients with incomplete records or with an incomplete follow-up period (i.e. less than 12 months since index hospitalization) were excluded.

We examined patients’ electronic and paper-based records and collected certain socio-demographic and clinical data concerning their first admission (i.e. index admission) during a 2-year period (2021-2022).

Based on the review of current literature, we determined the following criterion for the revolving door patient, also known as frequent service user (FSU): three or more hospitalizations during a 12-month period (6–8, 10, 11). As a result, patients were divided into two groups according to the frequent service user criteria: FSUs (i.e. patients with 3 or more hospitalizations in 12 months) and non-FSUs (i.e. patients with less than 3 hospitalizations in 12 months).

### 2.2 Variables

The following sociodemographic variables were retrieved: age, gender (male or female), residence location type (urban or rural), years of formal education, marital status (with partner or without partner), living situation (alone, with other – friends/family, in public residence or homeless), professional status (unemployed, retired, disability pension, student or employed).

The clinical data extracted was: length of index admission, illness duration (<5 years, 5-14 years, ≥15 years), presence of family psychiatric history, presence of psychiatric or medical comorbidities, number of hospitalizations during 12 consecutive months, type of admission (voluntary or involuntary), presence of alcohol use disorder, presence of substance use disorder, need of physical restraint, antipsychotic administration (oral or long-acting), undergoing clozapine treatment (yes or no), compliance to pharmacological treatment (yes or no), aggressive behavior evaluated using The Modified Overt Aggression Score (MOAS). MOAS is one of the subsequent versions of Overt Aggression Scale (OAS). OAS was elaborated in 1986 (25) and along with its modified versions is intended to assess aggression in different settings. MOAS covers four categories of aggressive behavior in the last week prior to administration: verbal aggression, aggression against property, autoaggression and physical aggression against others. Each category can be rated on a 5-point Likert scale. The final total score can reach a maximum of 40 and represents the sum of all weighted scores. Administration of MOAS does not require special qualification. Since its development, MOAS has been extensively used to assess aggressive behavior and was found to have satisfactory psychometric properties (25–28). In our hospital, MOAS is routinely administered on the day of admission and on the following days if necessary. The compliance to pharmacological treatment was assessed by the treating psychiatrist through interview with the patient and their family members or caregiver, as a standard procedure in our hospital. The presence of alcohol or substance use disorder was recorded by treating psychiatrist if the patient was actively using alcohol or psychoactive substances prior to hospitalization; patients in remission were not categorized as such.

### 2.3 Statistical analysis

IBM Statistical Package for Social Sciences (SPSS) version 26.0 was used for the statistical analysis in the present study. All tests were two-tailed and an alpha level of < 0.05 was considered statistically significant. Data were analyzed for distribution normality using the Kolmogorov-Smironov test. Continuous variables were expressed as mean and standard deviation (± SD), while categorical data were expressed as absolute (number) and relative (percentage) frequency. Statistical associations between categorical variables were performed using the Chi-square test after cross-tabulation. Continuous data from two independent groups were compared using the Mann-Whitney-U test. To identify the factors independently associated with the studied outcome (≥ 3 hospitalizations in 12 months), a multivariable binary logistic regression was performed. Variables were introduced in the regression analysis if: (1) they were associated in the descriptive analysis with the studied outcome, (2) are known to be associated with the studied outcome or (3) based on clinical reasoning. Therefore, the variables introduced in the regression were: age, gender, years of formal education, marital status, living situation, professional status, length of index admission, illness duration, presence of family psychiatric history, presence of psychiatric or medical comorbidities, number of hospitalizations during 12 consecutive months, type of admission, presence of alcohol use disorder, presence of substance use disorder, need of physical restraint, antipsychotic administration, undergoing clozapine treatment, compliance to pharmacological treatment, MOAS scores. A stepwise backward likelihood ratio method was used to remove factors if p value was > 0.1 and retain them in the model if p < 0.05 at each step in the regression. Results of the binary logistic regression were expressed as odds ratio (OR) together with the 95% confidence interval (95% CI), standard error and B value. Model’s goodness-of-fit was tested using the Hosmer-Lemeshow test. The model was considered well calibrated if p value > 0.05. Furthermore, the percentage of cases correctly predicted was reported, together with the Nagelkerke R square value.

Lastly, we reported the results in the model based on the TRIPOD (transparent reporting of a multivariable prediction model for individual prognosis or diagnosis) statement checklist (29) (**Supplementary Table S1**).

## 3 Results

**Figure 1:**
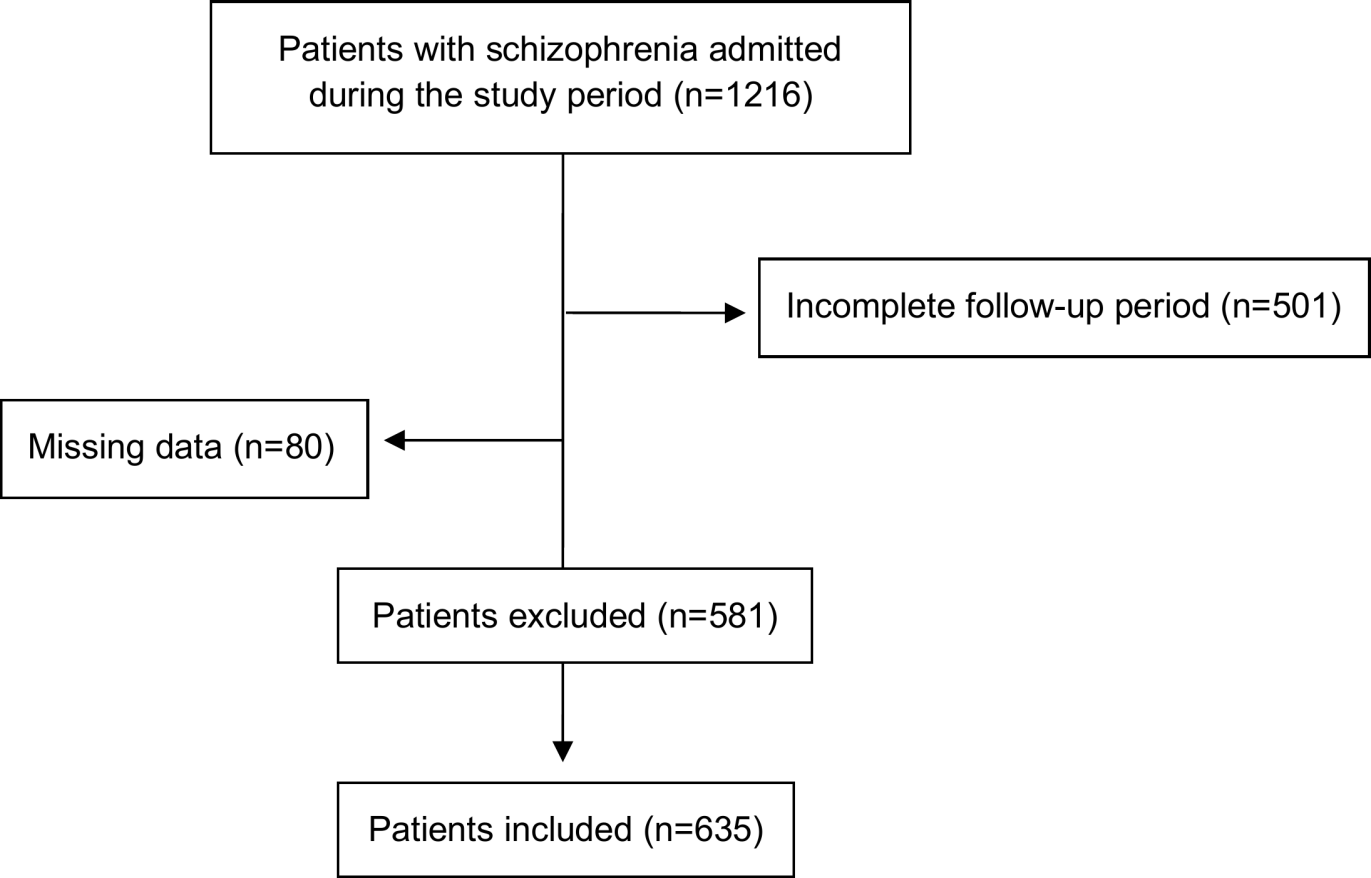
Flowchart of the study population.

Six hundred and thirty-five (n=635) patients, 364 (57.3%) females and 271 (42.7%) males, were included in the final analysis (**Figure 1**). Seventeen % (n=108) of the patients met the criterion for FSU and the mean ± SD age of the entire sample was 44.55±12.83. In both groups (i.e. FSUs and non-FSUs), most patients had a disability pension (67.6% and 68.9%, respectively), with no significant differences between them. Moreover, the majority of patients had an illness duration of 5 to 14 years, with no significant differences between FSUs and non-FSUs (p=0.416). Frequent re-hospitalized patients were more likely to have a psychiatric comorbidity (48,1% vs. 37.4%, p<0.05) but the two groups did not differ in terms of the presence of somatic comorbidities (37% vs. 63%, p>0.05). Alcohol use disorder and substance use disorder were associated with frequent use of inpatient psychiatric services (17.6% vs. 8.3%, p<0.01 and 14.8% vs. 4.2%, p<0.001, respectively). Regarding adherence to treatment, 63.9% of FSUs were non-adherent in comparison to 55% of non-FSUs, but the difference was not significant (p>0.05). All socio-demographic and clinical variables are presented in **Table 1**.

**Table 1:** Descriptive statistics for the socio-demographic and clinical variables of the study population.

	Total Sample	FSUs	Non-FSUs	p^1^
n	635	108(17.0%)	527(83.0%)	
Male gender	271 (42.7%)	66 (61.1%)	205 (38.9%)	0.000*
Age	44.55±12.83	41.78±11.63	45.06±13.04	0.015*
Urban residence	506	93(86.1%)	413(78.4%)	0.068
Years of education	11.6±3.25	11.29±3.44	11.65±3.21	0.480
Length of index hospitalization	17.99±16.66	15.94±10.59	18.35±17.57	0.315
Marital status				
With partner	116(18.3%)	16(14.8%)	100(19.0%)	0.308
Without partner	519(81.7%)	92(85.2%)	427(81.0%)	
Living situation				
Alone	150(23.6%)	27(25.0%)	123(23.3%)	0.184
With others (family/friends)	442(69.6%)	72(66.7%)	370(74.6%)	
In public residence	26(4.1%)	3(2.8%)	23(4.4%)	
Homeless	17(2.7%)	6(5.6%)	11(2.1%)	
Professional Status				
Unemployed	137(21.6%)	28(25.9%)	109(20.7%)	0.516
Retired	27(4.3%)	4(3.7%)	23(4.4%)	
Disability pension	436(68.7%)	73(67.6%)	363(68.9%)	
Student	4(0.6%)	0(0.0%)	4(0.8%)	
Employed	31(4.9%)	3(2.8%)	28(5.3%)	
Illness Duration				
<5 years	115(18.1%)	24(22.2%)	91(17.3%)	0.416
5-14 years	271(42.7%)	46(42.6%)	225(42.8%)	
≥15 years	249(39.2%)	38(36.2%)	211(40%)	
Family psychiatric history				
Yes	110(17.4%)	17(2.7%)	93(14.7%)	0.628
No	524(82.6%)	91(14.4%)	433(68.3%)	
Psychiatric comorbidity				
Yes	249(39.2%)	52(48.1%)	197(37.4%)	0.037*
No	386(60.8%)	56(51.9%)	330(62.6%)	
Somatic comorbidity				
Yes	266(41.9%)	40(37.0%)	226(42.9%)	0.262
No	369(58.1%)	68(63.0%)	301(57.1%)	
Type of index admission				
Voluntary	297(46.8%)	44(6.9%)	253(39.8%)	0.168
Involuntary	338(53.2%)	64(10.1%)	274(43.1%)	
Alcohol use disorder				
Yes	63(9.9%)	19(17.6%)	44(8.3%)	0.003*
No	572(90.1%)	89(82.4%)	483(91.7%)	
Substance use disorder				
Yes	38(6.0%)	16(14.8%)	22(4.2%)	0.000*
No	597(94.0%)	92(85.2%)	505(95.8%)	
MOAS				
Total score	2.60±0.213	2.90±5.642	2.52±5.286	0.916
Verbal aggression	0.49±0.036	0.50±0.925	0.48±0.893	0.949
Aggression against property	0.024±0.029	0.37±0.874	0.22±0.680	0.034
Autoaggression	0.11±0.022	0.04±0.235	0.12±0.600	0.287
Physical aggression	0.35±0.036	0.50±1.254	0.32±0.819	0.273
Physical restrain				
Yes	37(5.8%)	7(1.1%)	30(4.7%)	0.753
No	597(94.2%)	101(15.9%)	496(78.2%)	
Antipsychotic administration				
Oral	467(73.7%)	79(73.1%)	388(73.8%)	0.919
Long-acting injection	168(26.3%)	29(26.9%)	138(26.2%)	
Clozapine treatment				
Yes	96(15.1%)	11(10.2%)	85(16.1%)	0.116
No	519(84.9%)	97(89.8%)	422(83.9%)	
Compliance to treatment				
Yes	276(43.5%)	39(36.1%)	237(45.0%)	0.091
No	359(56.5%)	69(63.9%)	290(55.0%)	

FSU, frequent service user; MOAS, Modified Overt Aggression Scale; *p<0.05; ^1^Chi-square test or Mann-Whitney-U test.

For the studied outcome, 108 patients were classified as revolving door. The binary linear logistic regression model (R square Nagelkerke = 0.113, *p* < 0.001) was well calibrated (Hosmer-Lemeshow test: Chi-square = 14.53, p = 0.07) and showed that male gender, a shorter length of index hospitalization, higher MOAS – verbal aggression score, higher MOAS – physical aggression score and the presence of substance use disorder were predictors for frequent use of inpatient services (**Table 2**). The full regression model is presented in the **Supplementary Material** (**Supplementary Tables S2**; **Supplementary Table S3**).

**Table 2:** Independent predictors of frequent use of inpatient services – results of the binary linear logistic regression.

Overall cases correctly predicted: 83.1%, Nagelkerke R square = 0.113, Hosmer-Lemeshow (Chi-square = 14.53, p = 0.07)								
	B	S.E.	Wald	df	Sig.	Odds Ratio	95% CI for Exp(B)	
							Lower	Upper
Gender	-0.657	0.238	7.587	1	0.006	1.92	1.21	3.08
Length of index hospitalization	-0.019	0.009	3.897	1	0.048	0.982	0.964	1.000
MOAS – Verbal Aggression	-0.367	0.162	5.145	1	0.023	1.44	1.05	1.98
MOAS – Physical Aggression	0.286	0.138	4.327	1	0.038	1.331	1.017	1.744
Substance use disorder	-0.905	0.385	5.517	1	0.019	2.47	1.16	5.26
Constant	0.210	0.499	0.178	1	0.673	1.234		

MOAS, Modified Overt Aggression Scale.

## 4 Discussion

Increasing evidence suggests that the process of deinstitutionalization of psychiatric services has led to the emergence of the revolving door phenomenon. Therefore, this research sought to identify the socio-demographic and clinical correlates of this phenomenon in patients with schizophrenia in the largest psychiatric hospital in Romania. Our study demonstrated that frequent service use patients were usually males, younger, with a psychiatric comorbidity and with abuse or dependence of alcohol and illicit drugs. Moreover, male gender, a shorter hospitalization period, higher scores of verbal and physical aggression and illicit drugs use disorder have been found to be risk factors for frequent re-admissions. This schizophrenia-specific research adds to the limited evidence in the literature and, to our knowledge, is the first study in Romania to examine the revolving door phenomenon in psychiatry.

We found that male gender and younger age were associated with frequent rehospitalizations in schizophrenia patients. Current research has mixed results regarding the association of gender and age of schizophrenia patients with the revolving door phenomenon. Lerma-Carrillo et al. (2007) conducted a retrospective research on 209 adult patients admitted to a brief hospitalization unit. The authors compared the data of patients with only one hospitalization with patients admitted twice or more during a 12 month-period. They reported no differences regarding age or gender between the two groups (i.e. frequent users and non-frequent users) (30). Other studies have found that either male gender or younger age are associated with frequent use of inpatient psychiatric services, although it is worth noting that research by Lay et al. (2006) and Koparal et al. (2021) also included individuals with other primary psychotic disorders (i.e. schizoaffective disorder and other non-organic psychotic disorders and bipolar disorder and other non-organic psychotic disorders, respectively) (18, 19, 31). Given that women with schizophrenia have fewer hospitalizations and shorter hospital stays over their lifetime by comparison with men, the results showing that male gender is associated with and a risk factor for multiple readmissions are not surprising. Nonetheless, as Seeman M. V. (2019) noted, this may not always correspond to a better outcome since living in the community has its benefits, but also its downsides (i.e. poverty, health problems, stigma, isolation, etc.) compared to hospital environment (32).

In the current study, most of our revolving door patients (85.2%) were single, but this was not significantly different from non-frequent use patients (81%). Our results are in accordance with other relevant studies found in the literature (19, 31) but in opposition with those reported by Rabinowitz et al. (1995) who noted marriage to be a protective factor against revolving door phenomenon (33). People with schizophrenia usually have low rates of marriage compared with the general population in western countries (34). Moreover, in a study conducted by Mortensen and Eaton (1994) it was found that marital status was not a risk factor for readmission after 10 years of follow-up of patients with schizophrenia (35). Also, two recent reviews did not find compelling evidence supporting the role of marital status in psychiatric readmission (36, 37). Therefore, marital status may not be that relevant in terms of a particular pattern to access psychiatric services in our case. Also, the different research designs play an important role when comparing findings.

Consistent with literature (19, 31), we reported that living arrangements and professional status were not associated with the revolving door phenomenon in our cohort of patients. Lay et al. (2006) observed that homelessness and unemployment were associated with the longest time spent in hospital over a 5-year period and heavy use of inpatient services, respectively (18). Our results should be interpreted considering the country’s particularities. According to Eurostat, Romania had the highest share of people living in households owning in 2021 (95.3%) compared to the other European Union states (38). Also, most of young Romanians live with their parents (53.9% in 2022) (39), and this is reflected in our results as well. However, homelessness was reported to be a risk factor for readmission and emergency service visit in psychiatric patients (40), therefore future research is needed to make more conclusive statements. Our results indicate that most patients were receiving disability pension which reflects the lack of government-supported employment services for individuals with severe mental illnesses. Another study that included Romanian schizophrenia patients provide results in agreement with ours (41).

An important aspect revealed by the herein study is that the presence of psychiatric comorbidity is significantly associated with frequent use of hospital services but not a risk factor. The above findings contradict the study by Lerma-Carrillo et al. (2007) (30). In accordance with our results, Kessler et al. (2019) found a direct relationship between number of psychiatric diagnoses and number of ED visits and hospitalizations and mean length of stay (42). There are several explanations for these results. Firstly, an additional psychiatric diagnosis in schizophrenia patients is indicative of a more severe and intricated clinical picture (43). Therefore, these patients need complex care which cannot be adequately delivered in the community at times. Secondly, according to Kessler et al. (2019), overlooking the comorbid states or considering them as part of the schizophrenic heterogenous symptomatology might explain the association reported by our research (42).

Our research provides evidence on the association between alcohol use disorder and substance use disorder and multiple admissions to hospital. Moreover, it indicates that substance use disorder is a risk factor for the revolving door phenomenon. These results are in the lines of earlier literature that found that patients with a dual diagnosis (i.e. schizophrenia and substance use disorder or alcohol use disorder) were retained in the community for a shorter period (20, 21, 44, 45). The authors conclude that the benefit of antipsychotic medication on preventing readmission is reduced by substance abuse (44). Other researchers showed that significant differences did not exist (19, 30, 31), so findings are somehow contradictory. While Rømer Thomsen et al. (2018) found that use of cannabis increases the risk of readmission, Slaughter et al. (2017) reported results at difference with (20, 21). These contradictions stem from several factors such as different research designs (some studies define readmissions differently), variability in patient population (severity of illness, types of drugs investigated), and varying follow-up durations. Nevertheless, alcohol or substance use disorder have a major negative impact on the course of illness, including psychopathology and community functioning (46–48), which may explain the abovementioned results.

An observation to emerge from our data analysis was that verbal and physical aggression were predictors for the revolving door phenomenon in schizophrenia patients. A recent meta-analysis revealed that the pooled prevalence of aggression was 33.3% in patients with schizophrenia (49). According to Fresan et al. (2007), the relationship between aggression and hospital readmission is mediated by a vicious cycle where poor social support, lack of insight into illness, non-adherence to treatment, and relapse are contributory factors (50). In addition to positive symptoms, male gender, younger age, and substance abuse have strong connections with the occurrence of aggressive behavior in schizophrenia (51, 52). These results integrate well with those previously discussed and together assist in our understanding of the socio-demographic and clinical correlates of the revolving door phenomenon in patients with schizophrenia.

Our study has several limitations that need to be acknowledged. Firstly, our cohort consists of patients from a single center, therefore, limiting the generalizability of findings at a national level. Secondly, due to the retrospective collection of data from health records, we could not perform a fine-grained analysis of the variables (i.e. standard diagnosis and psychopathology assessments) or include other measures (e.g. suicidal scores, social support, patient’s contact with outpatient mental health services prior to hospital admission, etc.). Thirdly, the lack of consensus on the revolving door phenomenon definition affected the accuracy of comparison between studies. Lastly, our research was conducted during COVID-19 times which was shown to be associated with reduced inpatient admissions for schizophrenia even though specific restrictions and regulations regarding hospitalizations were not present in Romania during the research period (53).

## 5 Conclusion

Important conclusions drawn from this work include that characteristics such as male gender, younger age, substance or alcohol use disorder and proneness to physical or verbal aggressive behavior contribute to profiling the revolving door schizophrenia patient. These results call for an action plan addressing the problems of the mental health services organization. Different interventions based on the concept of transition of care between hospital and community services and on strengthening outpatient care were reported as possible solutions in reducing the revolving door phenomenon (54). Identifying patients in immediate need of such interventions is a key component for successful implementation of such programs.

## Data Availability

The raw data supporting the conclusions of this article will be made available by the authors, without undue reservation.
