# Supplementary material for: Factors associated with the revolving door phenomenon in patients with schizophrenia: results from an acute psychiatric hospital in Romania
Source: Front Psychiatry. 2025 Jan 24;15:1496750. doi: 10.3389/fpsyt.2024.1496750 (PMC11802556; doi:10.3389/fpsyt.2024.1496750)
Supplement: Supplementary file 1 [file Table1.docx]

Supplementary Material

# Supplementary Table 1. TRIPOD Checklist: Prediction Model Development

| **Section/Topic** | **Item** | **Checklist Item** | **Page** |
| --- | --- | --- | --- |
| **Title and abstract** | | | |
| Title | 1 | Identify the study as developing and/or validating a multivariable prediction model, the target population, and the outcome to be predicted. | 1 |
| Abstract | 2 | Provide a summary of objectives, study design, setting, participants, sample size, predictors, outcome, statistical analysis, results, and conclusions. | 1 |
| **Introduction** | | | |
| Background and objectives | 3a | Explain the medical context (including whether diagnostic or prognostic) and rationale for developing or validating the multivariable prediction model, including references to existing models. | 2 |
|  | 3b | Specify the objectives, including whether the study describes the development or validation of the model or both. | 2 |
| **Methods** | | | |
| Source of data | 4a | Describe the study design or source of data (e.g., randomized trial, cohort, or registry data), separately for the development and validation data sets, if applicable. | 2-3 |
|  | 4b | Specify the key study dates, including start of accrual; end of accrual; and, if applicable, end of follow-up. | 3 |
| Participants | 5a | Specify key elements of the study setting (e.g., primary care, secondary care, general population) including number and location of centres. | 3 |
|  | 5b | Describe eligibility criteria for participants. | 3 |
|  | 5c | Give details of treatments received, if relevant. | - |
| Outcome | 6a | Clearly define the outcome that is predicted by the prediction model, including how and when assessed. | 3 |
|  | 6b | Report any actions to blind assessment of the outcome to be predicted. | - |
| Predictors | 7a | Clearly define all predictors used in developing or validating the multivariable prediction model, including how and when they were measured. | 3 |
|  | 7b | Report any actions to blind assessment of predictors for the outcome and other predictors. | 3 |
| Sample size | 8 | Explain how the study size was arrived at. | 4 |
| Missing data | 9 | Describe how missing data were handled (e.g., complete-case analysis, single imputation, multiple imputation) with details of any imputation method. | 3 |
| Statistical analysis methods | 10a | Describe how predictors were handled in the analyses. | 3 |
|  | 10b | Specify type of model, all model-building procedures (including any predictor selection), and method for internal validation. | 3-4 |
|  | 10d | Specify all measures used to assess model performance and, if relevant, to compare multiple models. | 3-4 |
| Risk groups | 11 | Provide details on how risk groups were created, if done. | - |
| **Results** | | | |
| Participants | 13a | Describe the flow of participants through the study, including the number of participants with and without the outcome and, if applicable, a summary of the follow-up time. A diagram may be helpful. | 4 |
|  | 13b | Describe the characteristics of the participants (basic demographics, clinical features, available predictors), including the number of participants with missing data for predictors and outcome. | 4-6 |
| Model development | 14a | Specify the number of participants and outcome events in each analysis. | - |
|  | 14b | If done, report the unadjusted association between each candidate predictor and outcome. | - |
| Model specification | 15a | Present the full prediction model to allow predictions for individuals (i.e., all regression coefficients, and model intercept or baseline survival at a given time point). | Supplementary Table 2 |
|  | 15b | Explain how to the use the prediction model. | - |
| Model performance | 16 | Report performance measures (with CIs) for the prediction model. | 6 |
| **Discussion** | | | |
| Limitations | 18 | Discuss any limitations of the study (such as nonrepresentative sample, few events per predictor, missing data). | 7 |
| Interpretation | 19b | Give an overall interpretation of the results, considering objectives, limitations, and results from similar studies, and other relevant evidence. | 4,7 |
| Implications | 20 | Discuss the potential clinical use of the model and implications for future research. | 7 |
| **Other information** | | | |
| Supplementary information | 21 | Provide information about the availability of supplementary resources, such as study protocol, Web calculator, and data sets. | 8 |
| Funding | 22 | Give the source of funding and the role of the funders for the present study. | 8 |

# Supplementary Table 2. Full Regression Model – Method: Backward Likelihood Ratio Elimination.

Hosmer-Lemeshow: Chi-square = 14.53, p = 0.07

Nagelkerke R square: = 0.113, *p* < 0.001

Overall percentage of cases correctly predicted: 83.1%

|  | **Variables in the model** | B | S.E. | Wald | df | Sig. | OR | 95%CI Lower | 95%CI Upper |
| --- | --- | --- | --- | --- | --- | --- | --- | --- | --- |
| **Step 1** | **Age** | -,013 | ,013 | 1,004 | 1 | ,316 | ,987 | ,963 | 1,012 |
|  | **Gender (Male)** | -,573 | ,250 | 5,265 | 1 | ,022 | 1,77 | 1,09 | 2,9 |
|  | **Years of education** | -,013 | ,036 | ,134 | 1 | ,714 | ,987 | ,919 | 1,059 |
|  | **Illness duration (reference) (≥15y)** |  |  | ,030 | 2 | 0,98 |  |  |  |
|  | **Illness duration (1) (10-14 y)** | -,063 | ,412 | ,023 | 1 | ,879 | ,939 | ,419 | 2,106 |
|  | **Illness duration (2) (<5y)** | -,046 | ,291 | ,025 | 1 | ,875 | ,955 | ,540 | 1,689 |
|  | **Length of index hospitalization** | -,016 | ,010 | 2,845 | 1 | ,092 | ,984 | ,965 | 1,003 |
|  | **Marital status** | ,032 | ,329 | ,009 | 1 | ,923 | 1,033 | ,542 | 1,968 |
|  | **Living situation (reference)**  **Alone** |  |  | 1,718 | 3 | 0,63 |  |  |  |
|  | **Living situation (1)**  **With others (family/friends)** | -,450 | ,631 | ,509 | 1 | ,476 | ,638 | ,185 | 2,195 |
|  | **Living situation (2)**  **In public residence** | -,651 | ,602 | 1,170 | 1 | ,279 | ,522 | ,160 | 1,696 |
|  | **Living situation (3) Homeless** | -,896 | ,867 | 1,068 | 1 | ,301 | 2,45 | 0,45 | 13,33 |
|  | **Professional status (reference)** |  |  | 1,909 | 3 | ,592 |  |  |  |
|  | **Professional status (1) retired** | ,516 | ,676 | ,583 | 1 | ,445 | 0,6 | 0,16 | 2,25 |
|  | **Professional status (2) disability pension** | ,899 | ,872 | 1,064 | 1 | ,302 | 2,457 | ,445 | 13,557 |
|  | **Professional status (3) employed** | ,775 | ,650 | 1,423 | 1 | ,233 | 0,46 | 0,13 | 1,64 |
|  | **Psychiatric comorbidity** | -,215 | ,241 | ,791 | 1 | ,374 | ,807 | ,503 | 1,295 |
|  | **Somatic comorbidity** | ,196 | ,240 | ,668 | 1 | ,414 | 1,217 | ,760 | 1,948 |
|  | **Alcohol use disorder** | -,588 | ,337 | 3,046 | 1 | ,081 | 1,8 | 0,93 | 3,48 |
|  | **Substance use disorder** | -,810 | ,433 | 3,499 | 1 | ,061 | 2,25 | 0,96 | 5,26 |
|  | **Antipsychotic administration** | -,027 | ,264 | ,011 | 1 | ,918 | ,973 | ,580 | 1,632 |
|  | **Clozapine treatment** | ,266 | ,376 | ,498 | 1 | ,480 | 0,77 | 0,37 | 1,6 |
|  | **Noncompliance to treatment** | ,373 | ,252 | 2,194 | 1 | ,139 | 1,452 | ,886 | 2,380 |
|  | **MOAS verbal aggression** | -,362 | ,165 | 4,803 | 1 | ,028 | 1,44 | 1,04 | 1,98 |
|  | **MOAS Aggression against property** | ,274 | ,163 | 2,829 | 1 | ,093 | 1,316 | ,956 | 1,811 |
|  | **MOAS Autoaggression** | -,550 | ,398 | 1,912 | 1 | ,167 | 1,73 | 0,79 | 3,77 |
|  | **MOAS Physical aggression** | ,276 | ,144 | 3,679 | 1 | ,055 | 1,318 | ,994 | 1,748 |
|  | **Constant** | ,474 | 1,277 | ,138 | 1 | ,710 | 1,606 |  |  |

Variables removed (order of removal):

1. Illness duration (Overall score: 0,03, p = 0,985)
2. Marital status (Overall score: 0,008, p = 0,929)
3. Antipsychotic administration (Overall score: 0,008, p = 0,928)
4. Years of educatioon (Overall score: 0,150, p = 0,698)
5. Living situation (Overall score: 1,777, p = 0,62)
6. Professional status (Overall score: 1,915, p = 0,590)
7. Somatic comorbidity (Overall score: 0,402, p = 0,526)
8. Age (Overall score: 0,670, p = 0,413)
9. Psychiatric comorbidity (Overall score: 0,757, p = 0,384)
10. Clozapine treatment (Overall score: 0,837, p = 0,360)

# Supplementary Table 3. Final Model (Step 11)

|  | **Variables in the final model** | B | S.E. | Wald | df | Sig. | OR | 95% CI  Lower | 95% CI  Uper |
| --- | --- | --- | --- | --- | --- | --- | --- | --- | --- |
| **Step 11** | **Gender (Male)** | -,657 | ,238 | 7,587 | 1 | ,006 | 1,93 | 1,21 | 3,08 |
|  | **Length of index hospitalization** | -,019 | ,009 | 3,897 | 1 | ,048 | ,982 | ,964 | 1,000 |
|  | **Alcohol use disorder** | -,620 | ,323 | 3,700 | 1 | ,054 | 1,86 | 0,98 | 3,5 |
|  | **Substance use disorder** | -,905 | ,385 | 5,517 | 1 | ,019 | 2,47 | 1,16 | 5,26 |
|  | **Non-compliance** | ,402 | ,243 | 2,741 | 1 | ,098 | 1,495 | ,929 | 2,408 |
|  | **MOAS verbal aggression score** | -,367 | ,162 | 5,145 | 1 | ,023 | 1,44 | 1,05 | 1,98 |
|  | **MOAS Aggression against property** | ,287 | ,161 | 3,168 | 1 | ,075 | 1,332 | ,971 | 1,827 |
|  | **MOAS Autoaggression** | -,531 | ,382 | 1,933 | 1 | ,164 | 1,7 | 0,8 | 3,6 |
|  | **MOAS Physical aggression** | ,286 | ,138 | 4,327 | 1 | ,038 | 1,331 | 1,017 | 1,744 |
|  | **Constant** | ,210 | ,499 | ,178 | 1 | ,673 | 1,234 |  |  |
